# INCIDENTAL HEPATIC STEATOSIS IDENTIFIED ON ULTRASOUND IN PATIENTS UNDERGOING CHOLECYSTECTOMY: HIGH PREVALENCE AND INSUFFICIENT INVESTIGATIVE AND CLINICAL MANAGEMENT

**DOI:** 10.1590/S0004-2803.24612024-118

**Published:** 2025-05-02

**Authors:** Heloísa Mello TRAPP, Paulo André Bispo MACHADO-JÚNIOR, Silvania Klug PIMENTEL

**Affiliations:** 1 Universidade Federal do Paraná, Faculdade de Medicina, Curitiba, PR, Brasil.; 2 Hospital do Trabalhador, Serviço de Cirurgia Geral, Curitiba, PR, Brasil.

**Keywords:** Hepatic steatosis, non-alcoholic fatty liver disease, cholecystectomy, incidental findings, metabolic dysfunction-associated steatotic liver disease, Esteatose Hepática, hepatopatia gordurosa não alcoólica, colecistectomia, achados incidentais, doença hepática gordurosa associada à disfunção metabólica

## Abstract

**Background:**

Steatotic liver disease (SLD) affects about 1 billion people globally, making its proper management essential to prevent progression to more severe stages.

**Objective:**

The aim of this study was to evaluate medical management concerning hepatic steatosis incidentally identified by ultrasound in patients undergoing elective cholecystectomy.

**Methods:**

This observational, cross-sectional, and retrospective study included patients aged 18 years or older who underwent elective cholecystectomy at *Hospital do Trabalhador*, in Curitiba/PR, between 2018 and 2022. Patients with external ultrasound reports or incomplete data in their medical records were excluded. Medical records, laboratory tests, and ultrasound reports were analyzed to evaluate the prevalence of steatosis in these patients.

**Results:**

The study sample consisted of 355 patients, and 103 (29.01%) of them presented steatosis on ultrasound. Older age (*P*=0.0022), male sex (*P*=0.03009), higher body mass index (*P*<0.001), obesity (*P*<0.001), hypertension (*P*<0.001), dyslipidemia (*P*=0.0072), and elevated levels of oxaloacetic and pyruvic aminotransferases (*P*=0.02112) were associated with the presence of this finding. No action was taken regarding the presence of steatosis in 60.19% of patients. Approximately 39.81% had the finding recorded in their medical records, 6.80% received lifestyle change counseling, and 4.85% were investigated for the stage of steatosis.

**Conclusion:**

A significant prevalence of hepatic steatosis was incidentally identified in the ultrasound of patients undergoing cholecystectomy. However, the approach to this finding was insufficient, highlighting the need for substantial improvements on its management and investigation.

## HIGHLIGHTS

• Hepatic steatosis was incidentally identified in 29.01% of patients undergoing elective cholecystectomy.

• Older age, male sex, high BMI, obesity, hypertension, and dyslipidemia were significantly associated with the presence of steatosis.

• The approach to hepatic steatosis was insufficient, highlighting the need for improved management and investigation.

## RESUMO

**Contexto:**

A doença hepática esteatótica (DHE) afeta cerca de 1 bilhão de pessoas globalmente, sendo fundamental seu manejo adequado para evitar sua progressão para estágios mais graves.

**Objetivo:**

O objetivo do estudo foi avaliar a conduta médica referente à esteatose hepática identificada incidentalmente no ultrassom de pacientes submetidos à colecistectomia eletiva.

**Métodos:**

Estudo observacional, transversal e retrospectivo, que incluiu pacientes com idade igual ou superior a 18 anos submetidos à colecistectomia eletiva no Hospital do Trabalhador, em Curitiba/PR, entre 2018 e 2022. Foram excluídos pacientes com ultrassonografia externa e com dados incompletos no prontuário. Foram analisados prontuários, exames laboratoriais e laudos ultrassonográficas, com avaliação da prevalência da esteatose nesses pacientes.

**Resultados:**

A amostra do estudo foi composta por 355 pacientes, e 103 (29,01%) deles apresentaram esteatose ao ultrassom. Idade elevada (*P*=0,0022), sexo masculino (*P*=0,03009), índice de massa corporal elevado (*P*<0,001), obesidade (*P*<0,001), hipertensão arterial (*P*<0,001), dislipidemia (*P*=0,0072) e maiores níveis de transaminases oxalacética e pirúvica (*P*=0,02112) associaram-se à presença desse achado. Não houve conduta em relação à presença de esteatose em 60,19% dos pacientes. Cerca de 39,81% tiveram o achado anotado em seu prontuário, 6,80% receberam orientações sobre mudanças no estilo de vida e 4,85% foram investigados quanto ao estágio da esteatose.

**Conclusão:**

Uma prevalência notável de esteatose hepática foi identificada incidentalmente no ultrassom de pacientes submetidos à colecistectomia. Contudo, a abordagem desse achado revelou-se insuficiente, com necessidade de melhorias substanciais no seu manejo e investigação.

## INTRODUCTION

Steatotic liver disease (SLD) is the leading cause of chronic liver disease in Western countries^1^, affecting 25 to 30% of adult individuals^2^ and approximately one billion people worldwide^3^. Projections indicate that the disease will become the primary indication for liver transplantation in the coming decades^4^. SLD is the overarching term for conditions characterised by abnormal lipid accumulation in the liver (hepatic steatosis). It encompasses metabolic dysfunction-associated steatotic liver disease (MASLD), alcohol-related liver disease (ALD), the overlap between MASLD and ALD (MetALD), and rare causes of liver steatosis^5^.

SLD is a progressive, clinically and pathologically silent entity. It encompasses a spectrum of conditions including uncomplicated steatosis, steatohepatitis, and fibrosis^4^, with potential progression to more severe forms such as cirrhosis, hepatocellular carcinoma, and liver failure. Additionally, it has the potential to trigger extrahepatic complications such as cardiovascular diseases, renal failure, and sleep apnea^6^ ^,^ ^7^.

The primary underlying etiology of SLD is MASLD^8^ ^,^ ^9^, which is characterized by lipid accumulation in the liver exceeding 5%, confirmed by imaging or biopsy, in the presence of at least one associated cardiometabolic risk factor: overweight or obesity, abdominal circumference greater than 94 cm for men and 80 cm for women, prediabetes or type 2 diabetes mellitus, prehypertension or systemic arterial hypertension, and/or dyslipidemia, and in the absence of excessive alcohol consumption (≥30 g daily for men and ≥20 g daily for women) or other chronic liver diseases^8^ ^,^ ^10^ ^,^ ^11^. 

Due to the high prevalence of MASLD, hepatic steatosis is frequently detected incidentally during imaging exams^12^. Moreover, its complications and association with cardiovascular diseases and metabolic syndrome make steatosis a finding of significant clinical and public health importance^13^.

Thus, given the high morbidity and costs associated with this condition, early diagnosis and appropriate management of hepatic steatosis by the medical team are imperative^14^. In this context, the primary objective of the study was to evaluate the management of hepatic steatosis findings in ultrasound examinations of patients undergoing elective cholecystectomy. 

## METHODS

This was an observational, cross-sectional, and retrospective study conducted at the *Hospital do Trabalhador* in Curitiba (PR). The study protocol was approved by the institution’s Research Ethics Committee, under protocol number 62460322.7.0000.5225.

Electronic medical records, laboratory tests, and ultrasound examinations of patients who underwent elective cholecystectomy at the hospital over a five-year period, from January 1, 2018, to December 31, 2022, were analyzed. Patients aged 18 years or older at the time of surgery were included. Exclusion criteria consisted of patients who had ultrasound examinations performed at external institutions and thus lacked official examination reports, and patients with insufficient medical record data for the study.

The following variables were assessed: age, gender, body mass index (BMI, calculated by dividing the patient’s weight in kilograms by the square of their height in meters), overweight (BMI between 25 and 29.9 kg/m²), obesity (BMI of 30 kg/m² or higher), type 2 diabetes mellitus, systemic arterial hypertension (SAH), dyslipidemia, smoking, alcohol use, use of potentially hepatotoxic medications, elevation of aminotransferases (aspartate aminotransferase [AST] and/or alanine aminotransferase [ALT]), elevation of alkaline phosphatase (ALP) and/or gamma-glutamyl transferase (GGT), elevation of bilirubins (total, direct, and/or indirect), intraoperative and postoperative complications, hospital length of stay, indications for surgery (classified as symptomatic cholelithiasis, gallbladder polyp, oligosymptomatic cholelithiasis, biliary sludge, and gallbladder adenomyomatosis), and presence or absence of hepatic steatosis.

The reference values for laboratory tests were based on those used by the hospital’s laboratory: AST between 13 U/L and 40 U/L, ALT between 10 U/L and 49 U/L, ALP between 45 U/L and 129 U/L, GGT less than 73 U/L for men and less than 38 U/L for women, total bilirubin between 0.2 mg/dL and 1.3 mg/dL, direct bilirubin less than 0.3 mg/dL, and indirect bilirubin less than 1.1 mg/dL. Hepatic steatosis was identified based on the description and report of abdominal ultrasound performed by the radiologist during the preoperative investigation for cholecystectomies. The radiologist, blind to the study, classified the steatosis according to the Saadeh et al. classification, graded as: grade I (mild, increased hepatic echogenicity with normal visualization of intrahepatic vessels and diaphragm), grade II (moderate, increased hepatic echogenicity with blurring of visualization of intrahepatic vessels and diaphragm), and grade III (severe, increased hepatic echogenicity with non-visualization of intrahepatic vessels, diaphragm, and posterior liver region)^15^.

Patients were divided into groups based on the presence or absence of hepatic steatosis detected by ultrasound. Variables compared between the two groups included age (mean and standard deviation), gender, BMI, overweight, obesity, diabetes mellitus, systemic arterial hypertension, dyslipidemia, smoking, alcohol use, use of hepatotoxic medications, elevation of liver aminotransferases (AST and/or ALT), elevation of canalicular enzymes (GGT and/or ALP), elevation of bilirubins (total, direct, and/or indirect), intraoperative complications, postoperative complications, and hospital length of stay.

In the group of patients with hepatic steatosis detected by ultrasound, the presence or absence of medical interventions by the healthcare team was assessed. Interventions considered included: documentation in the medical record, evaluation of the Fibrosis-4 Index (FIB-4), lifestyle modification recommendations (diet, physical exercise, and weight loss), liver biopsy, and referral to a hepatology service. The FIB-4 is a liver fibrosis index derived from the patient’s age, AST, ALT, and platelet levels.

Data were tabulated using Microsoft Excel®. In the descriptive analysis, categorical variables were expressed as absolute numbers and percentages. For numerical variables, the mean and standard deviation were calculated. In the inferential statistical comparison of variables, the Pearson Chi-Square test and Fisher’s Exact test were used for categorical variables, and the Mann-Whitney test was employed for continuous variables. *P*-values less than 0.05 were considered statistically significant. Data were analyzed using the R statistical software (R Core Team, 2022) version 4.2.1^16^.

## RESULTS

During the analyzed period, 1,374 elective cholecystectomies were performed at the hospital, of which 1,372 were performed on patients aged 18 years or older. Of these 1,372 patients who met the inclusion criteria, 1,017 patients were excluded: 1,016 for not having an ultrasound performed at the hospital and 1 for having insufficient data in the medical records.

The study included 355 patients, whose ages ranged from 20 to 88 years (mean age of 48.6±15.2 years). Of the 355 patients, 264 (74.36%) were female and 91 (25.63%) were male. The main indication for surgery was symptomatic cholelithiasis in 319 patients (89.86%). Eighteen patients (5%) underwent surgery for gallbladder polyps, nine patients (2.54%) for oligosymptomatic cholelithiasis, five patients (1.4%) for biliary sludge, and four patients (1.13%) for gallbladder adenomyomatosis. Among the patients analyzed, 103 (29%) had hepatic steatosis on ultrasound, with the most prevalent presentations being grade I (n=55; 15.49%) and grade II (n=42; 11.83%) steatosis (Table 1).

**TABLE 1:** Characteristics of patients undergoing elective cholecystectomy.

Variable	N=355	%
Age (years), mean ± standard deviation	48.6±15.2	
Gender		
Female	264	74.37
Male	91	25.63
Indication for surgery		
Symptomatic cholelithiasis	319	89.86
Gallbladder polyp	18	5.07
Oligosymptomatic cholelithiasis	9	2.54
Biliary sludge	5	1.40
Gallbladder adenomyomatosis	4	1.13
Presence of hepatic steatosis	103	29.01
Grade I	55	15.49
Grade II	42	11.83
Grade III	6	1.69

Table 2 shows the comparison between the groups with and without hepatic steatosis on ultrasound in the studied population. Patients with hepatic steatosis had a higher average age (52.17±13.88) compared to patients without hepatic steatosis (47.07±15.52) (*P*=0.0022). Additionally, the percentage of males in the steatosis group (33.65%) was higher than in the non-steatosis group (22.22%) (*P*=0.03009). The average BMI in the steatosis group was 32.37 kg/m² ± 5.29, higher than that of the non-steatosis group, whose average BMI was 27.12 kg/m² ± 4.86 (*P*=0.0022). 

**TABLE 2:** Comparison between patients with and without hepatic steatosis on ultrasound.

Variable	With steatosis on ultrasound (n=103)	%	No steatosis on ultrasound (n=252)	%	P-value
Age (years), mean ± standard deviation	52.17±13.88		47.07±15.52		0.0022a
Gender					
Female	68	65.38	196	77.78	0.03009b
Male	35	33.65	56	22.22	
BMI (kg/m²)	32.39±5.29		28.09±4.86		<0.001a
Overweight	27	32.93	93	42.86	0.1525b
Obesity	54	62.07	66	30.28	<0.001b
Diabetes mellitus	18	17.48	24	9.52	0.05433b
Hypertension	56	54.37	70	27.78	<0.001b
Dyslipidemia	23	22.33	27	10.71	0.0072b
Smoking	13	12.62	44	17.46	0.3332b
Alcoholism					
Currently an alcoholic	2	1.94	5	1.98	0.1045c
Ex-alcoholic	5	4.85	3	1.19	
Non-alcoholic	96	93.2	244	96.83	
Use of potentially hepatotoxic medications	20	19.42	45	17.86	0.8463c
Elevated aminotransferases levels¥	17	34.69	15	16.13	0.02112b
Elevated canalicular levels£	18	40.9	34	37.36	0.8351b
Elevated bilirubin levelsˆ	3	6.98	10	10.75	0.7546c
Intraoperative complications	2	1.94	5	1.98	1.00c
Complications after discharge	5	4.85	7	2.78	0.3406c
Length of hospital stay (days)	1.22±0.77		1.2±0.75		0.9836a

BMI: body mass index. ^¥^ Defined by oxaloacetic aminotransferase greater than 40U/l and/or pyruvic aminotransferase greater than 49U/l. Absence of aminotransferase data for 54 patients with steatosis and 159 patients without steatosis; ^£^Defined by alkaline phosphatase greater than 129U/l and/or gamma glutamyl transferase greater than 73U/l for men and greater than 38U/l for women. Absence of canalicular data for 59 patients with steatosis and 161 patients without steatosis; ^ˆ^Defined by total bilirubin greater than 1.3 mg/dL, direct bilirubin greater than 0.3 mg/dl and/or indirect bilirubin greater than 1.1 mg/dL. Absence of bilirubin data for 60 patients with steatosis and 159 patients without steatosis; ^a^Mann-Whitney test; ^b^Pearson ‘s Chi-Square test; ^c^ Fisher ‘s Exact Test. Variables that presented missing data for some patients were analyzed based on the total data available for the variable in question.

Moreover, a statistically significant relationship was observed between hepatic steatosis and obesity (*P*<0.001), systemic arterial hypertension (*P*<0.001), dyslipidemia (*P*=0.0072), and altered liver enzymes (AST and/or ALT) (*P*=0.02112). No statistical significance was found for the variables of being overweight, diabetes mellitus, smoking, alcohol consumption, use of medication with potential hepatotoxicity, canalicular alterations, bilirubin alterations, intraoperative complications, postoperative complications after hospital discharge, or length of hospital stay.

In the analysis of the 103 patients with steatosis on ultrasound, no action was taken for 62 of them (60.19%). For the patients where some action was taken, the main action was making a note in the medical record for 41 patients. In only eight patients were actions taken beyond noting in the medical record: for seven (6.80%) of them, lifestyle change counseling was provided, and for one (0.97%), a liver biopsy was requested. 

**FIGURE 1:**
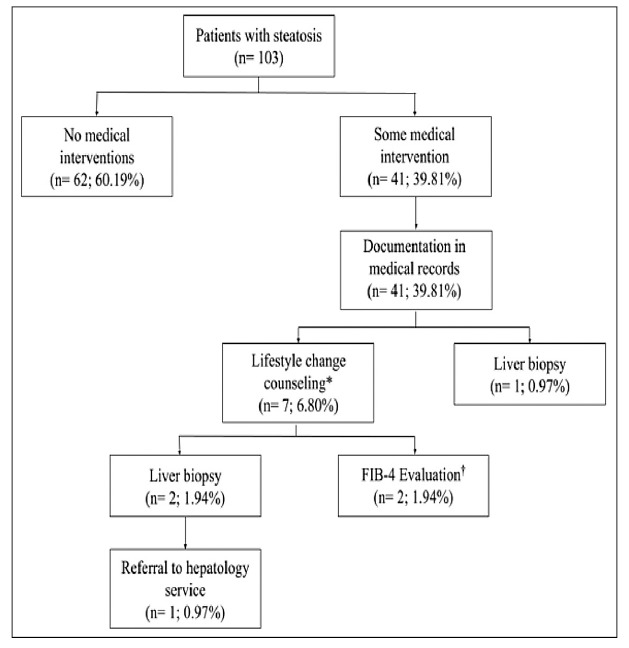
Management in patients with hepatic steatosis on ultrasound.

Of the seven patients who had both a medical record note and lifestyle change counseling, for two (1.94%) the FIB-4 score was calculated, resulting in 0.91(U/L)(109/L)/(U/L)(years²) and 0.96(U/L)(109/L)/(U/L)(years²), both classified as “low risk.” For another two patients (1.94%), a liver biopsy was performed. One patient from this group who underwent a liver biopsy was referred to a hepatology service (0.97%) (Figure 1).

FIB-4, Fibrosis Index-4. *Includes dietary, exercise and weight loss counseling; † Obtained from patient age, oxaloacetic aminotransferase, pyruvic aminotransferase and platelet levels.

## DISCUSSION

SLD is the fastest-growing liver disease in the world, with distribution varying according to geographic region, ethnicity, socioeconomic factors and lifestyle^17^ ^,^ ^18^. In 2016, its global prevalence was 25%, progressing to 30% in 2019^19^. Brazil is one of the countries with the highest prevalence of SLD worldwide^6^ ^,^ ^17^, reaching 35.2% of the general population^20^. In the present study, of the 355 patients who underwent cholecystectomy, 29% presented hepatic steatosis on the abdominal ultrasound performed preoperatively. This prevalence is lower than that found in previous studies in Brazil (35.2%)^20^ but similar to global levels (30%)^19^. 

Although its pathogenesis is not yet fully understood, SLD is strongly associated with components of metabolic syndrome, such as obesity, insulin resistance, type 2 diabetes mellitus, hypertension, and dyslipidemia^2^ ^,^ ^21^. In this study, most patients with steatosis had at least one cardiometabolic risk factor, especially high weight, suggesting an important metabolic component in the pathogenesis of hepatic steatosis in these patients. Moreover, these same cardiometabolic components are also risk factors associated with cholelithiasis^2^ ^,^ ^21^ ^,^ ^22^, the main indication for cholecystectomy^23^. Additionally, clinical studies and systematic reviews that examined the association between these two conditions demonstrated a bidirectional relationship between them: just as SLD is an independent risk factor for cholelithiasis, cholelithiasis is also a risk factor for SLD, and is even associated with greater disease severity^2^. However, despite the increased risk of patients with cholelithiasis developing SLD, there was no higher prevalence of hepatic steatosis in patients undergoing cholecystectomy compared to the general population, either globally or in Brazil^19^ ^,^ ^20^.

In this study, advanced age and male gender were related to the presence of hepatic steatosis on ultrasound. Increased age is associated with higher rates of SLD and liver fibrosis^6^. Regarding gender, studies conducted with populations in Thailand and Sri Lanka indicated a 4.3 times higher prevalence in females compared to males, whereas studies in the United States, China, and Spain revealed a higher prevalence of steatosis in men^6^. Additionally, our study demonstrated that elevated BMI, obesity, hypertension, and dyslipidemia were factors that showed a statistically significant association with the finding of hepatic steatosis, in line with previous studies that also demonstrated a relationship between steatosis and these factors of Metabolic Syndrome^1^ ^,^ ^2^ ^,^ ^6^ ^,^ ^17^ ^,^ ^24^. The condition of obesity is associated with more severe forms of steatosis, such as steatohepatitis, cirrhosis, and hepatocellular carcinoma, increasing mortality in these patients^25^. 

Despite this, our results did not show a statistically significant association between the presence of hepatic steatosis on ultrasound and type 2 diabetes mellitus, contrary to previous studies that positively associated these two conditions^2^ ^,^ ^4^ ^,^ ^24^, which even identified insulin resistance as the central component of SLD^2^ ^,^ ^24^. Furthermore, higher levels of liver aminotransferases (AST and/or ALT) were present in patients with hepatic steatosis, in accordance with Kutaiba et al.^13^. This finding reveals higher levels of hepatocyte damage and apoptosis in these patients due to oxidative stress and the inflammatory process in the liver promoted by fat deposition in the organ^1^.

According to the American Association for the Study of Liver Diseases (AASLD)^5^, the initial evaluation of these patients should include the investigation of metabolic comorbidities, alcohol use, and exclusion of other causes of liver diseases. In patients with metabolic risk factors, it is recommended to calculate the risk of fibrosis development using the FIB-4 score. Values equal to or greater than 1.3 (109/L)/(U/L)(years²), which indicate intermediate or high risk of fibrosis, suggest referring the patient to a hepatology center for elastography. If elastography shows advanced fibrosis, a liver biopsy is indicated^10^ ^,^ ^26^. In Brazil, the Brazilian Guideline for Steatotic Liver Disease Associated with Metabolic Dysfunction, prepared by the Brazilian Society of Endocrinology and Metabolism (SBEM), in partnership with the Brazilian Association for the Study of Obesity and Metabolic Syndrome (ABESO) and the Brazilian Society of Hepatology (SBH), also recommends that steatotic patients with obesity or overweight be investigated for other causes of liver disease and stratified for liver fibrosis, with periodic reassessments of disease progression and response to treatment^11^. Such investigation was found to be infrequent in our study, as the stage of steatosis was assessed in only five patients, with the FIB-4 score calculated for two patients and liver biopsy performed in three patients, with only one being referred to a hepatology center. The lack of steatosis investigation was also observed in the study by Wright et al., where none of these three investigative measures were performed on any of the 127 evaluated patients^12^.

In our study, 60.19% of patients with steatosis did not have the finding documented in their medical records. This lack of documentation can lead to problems in the medical care of these patients, subjecting them to inadequate follow-up. Moreover, clinically significant cases of steatohepatitis and advanced fibrosis may go unnoticed and progress without proper treatment or cancer screening^12^.

The goals of treatment and management of patients with SLD are to decrease the morbidity and mortality related to the disease and to reverse steatohepatitis and fibrosis, or at least halt its progression to more advanced stages. Management should also reduce cardiovascular risk and promote cardiometabolic health^27^.

The initial treatment recommended for steatosis primarily involves lifestyle changes, such as physical exercise, dietary re-education, and control of weight and other associated metabolic factors^8^ ^,^ ^28^ ^,^ ^29^. In this sense, the incidental finding of hepatic steatosis on imaging exams is an opportunity for the medical team to address the disease^30^, through assessments and recommendations that could change the disease’s outcome. In the sample of the present study, lifestyle improvement counseling was given to only seven patients with the finding of steatosis, revealing a lack of clinical approach to these patients. Thus, our results demonstrated that, despite the severity of SLD and its potential complications, the presence and significance of the disease are often neglected by the attending medical team.

Important limitations should be noted regarding the present study. The first relates to its timeframe, which included the 2019 coronavirus pandemic, a period during which elective surgeries were limited, reducing the study sample. Additionally, a significant portion of patients who underwent cholecystectomy had their ultrasound exams performed in external institutions and were, therefore, excluded from the study. Moreover, although ultrasound is the method of choice for detecting hepatic steatosis due to its wide availability and low cost, it is operator-dependent^31^, which may influence the detection of steatosis. Furthermore, the prevalence of hepatic steatosis found in the study does not necessarily reflect the general population but rather a specific group of patients who underwent elective cholecystectomy at the hospital during the study period.

## CONCLUSION

A considerable prevalence of hepatic steatosis was incidentally identified in ultrasound exams performed on patients undergoing elective cholecystectomy. However, the clinical approach in response to this imaging finding was insufficient, with low rates both in the investigation of steatosis and in the provision of guidance regarding lifestyle changes.
